# Prevalence and antimicrobial resistance of Enterobacterales bacteria isolated from retail food in Poland

**DOI:** 10.2478/jvetres-2025-0055

**Published:** 2025-10-07

**Authors:** Magdalena Łopatek, Edyta Denis

**Affiliations:** Department of Microbiology of Food and Feed, National Veterinary Research Institute, 24-100 Puławy, Poland

**Keywords:** Enterobacterales, retail food, prevalence, antimicrobial resistance, multidrug resistance

## Abstract

**Introduction:**

The prevalence of potentially pathogenic microorganisms in different foods is widely researched. The aim of this study was to investigate the prevalence and antimicrobial resistance of selected Enterobacterales species isolated from retail food of animal origin in Poland.

**Material and Methods:**

Cold cuts, cold-smoked fish and cheeses making 194 samples were tested with the ISO horizontal method for the detection of *Enterobacteriaceae*, and then Enterobacterales isolates were identified using matrix-assisted laser desorption/ionisation–time of flight mass spectrometry (MALDI-TOF). The isolates’ antimicrobial susceptibility was determined using the minimal inhibitory concentration method.

**Results:**

Enterobacterales were detected in 159 (82.0%) samples, from which 226 bacterial isolates were recovered. Six bacterial species accounted for 65.9% of Enterobacterales isolates: *Escherichia coli* (n = 41), *Enterobacter cloacae* (n = 26), *Hafnia alvei* (n = 25), *Citrobacter* spp. (n = 20), *Serratia liquefaciens* (n = 20) and *Klebsiella oxytoca* (n = 17). The isolated *E. coli* strains showed low resistance to seven antimicrobials. *E. cloacae* isolates were mostly resistant to ampicillin (76.9%) and azithromycin (38.5%), *S. liquefaciens* to colistin (100%) and *H. alvei* strains to colistin (96.0%) and ampicillin (60.0%). The majority of *K. oxytoca* isolates (70.6%) were resistant to ampicillin, whereas only five *Citrobacter* isolates were. Twenty of the total pool of isolates (8.8%) were defined as multidrug resistant.

**Conclusion:**

Retail food of animal origin can be contaminated with various species of Enterobacterales, including microorganisms pathogenic to humans as well as others resistant to commonly used antimicrobials.

## Introduction

Enterobacterales are Gram-negative, mostly non-pathogenic or opportunistic bacteria that are part of the gastrointestinal microflora of humans and animals (19). They can include pathogenic species (*e.g*. of the *Escherichia, Salmonella, Shigella, Enterobacter, Klebsiella* and *Citrobacter* genera) responsible for foodborne infections which are some of the most serious health problems, especially in persons with compromised immunity and in the elderly, children or pregnant women (1, 15, 16, 22). Furthermore, extraintestinal infections linked to enterobacterial species are also often observed, mainly as a result of bacterial translocation from the gastrointestinal tract to the blood or lymph nodes. These infections can trigger generalised disease processes such as bloodstream infections, chronic kidney disease, haemolytic uraemic syndrome, spleen infections, osteomyelitis and meningitis (16, 25). Infections with Enterobacterales are usually treated with β-lactam antibiotics including penicillins, carbapenems and cephalosporins, and antimicrobial resistance to these antibiotics is a major current problem in medicine (19, 26), threatening public health and impacting the economy negatively. Antimicrobial agents are not always effective in the treatment of infections because in some instances bacteria have acquired resistance to all available antibiotics (5, 9). Acquisition of resistance genes is likely for Enterobacterales with their wide environmental dispersion, and these bacteria can quickly transmit them to other microorganisms (39).

Recently, a rapid increase in the number of microorganisms resistant to antimicrobial agents has been observed, which is related to the common use of antimicrobials, especially in human and veterinary medicine, livestock farming and crop production (9, 33). Antimicrobial-resistant bacteria originating from the environment and animals may be the source of resistance mechanisms for components of the human microflora. In addition, food such as meat and meat products, milk and dairy products, fish, fruit and vegetables is an important reservoir of bacteria with antimicrobial resistance genes (5, 24, 37). Important mechanisms of Enterobacterales’ resistance to antimicrobials involve the enzymes they produce such as β-lactamases with an extended spectrum of action, metallo-β-lactamases and cephalosporinases, which are the main defence mechanisms of microorganisms against β-lactam antibiotics (23, 26). The ability of Enterobacterales to accumulate, disseminate and express antimicrobial resistance mechanisms is also a very important public health issue. Countering the risk of the rapid spread of antimicrobial-resistant bacteria, especially multidrug-resistant (MDR) strains in the human environment, requires the implementation of monitoring and control procedures.

Moreover, a major risk for public health constitutes ready-to-eat food, including retail food of animal origin intended for direct human consumption. In this type of food pathogenic microorganisms such as *Listeria monocytogenes, Staphylococcus aureus* but also various species of Enterobacterales can easily survive and cause foodborne illness (29). Food of this type is susceptible to microbiological hazards to an extent depending on the hygienic conditions, food preparation method and the conditions of distribution and storage of the products (38). In recent years, the consumption of this kind of food has significantly increased, especially in developed countries as a result of lifestyle changes and its greater availability. Consumption carries the risk of foodborne diseases, which poses a serious threat to consumer health (29, 38).

The aim of the present study was to investigate the prevalence and antimicrobial resistance of selected species of Enterobacterales isolated from retail food of animal origin produced in Poland. This study does not mainly focus on *Salmonella, Klebsiella* or Shiga toxinproducing *E. coli* species, which are the Enterobacterales with widely reported human pathogenicity in the literature. It also examines non-pathogenic members of the Enterobacterales order, which serve as useful indicator organisms providing information on general food hygiene.

## Material and Methods

### Sample collection

A total of 194 retail food samples were examined including 96 samples of cold cuts, 46 samples of cold-smoked fish and 52 samples of cheeses (comprising 28 samples of goat cheese, 16 of cow’s milk cheese and 8 of sheep cheese) (Table 1). All products were purchased in grocery shops, wholesalers or organic farms located in various regions of Poland. The cold cuts and cheeses were produced in Poland from Polish raw materials in small local plants and farms, mainly according to traditional recipes. The cold-smoked fish were produced in fish-processing plants located in five voivodeships in Poland, and the raw material used for their production originated mostly from Norway (Table 1). The samples were transported to the laboratory under refrigeration conditions (1–8°C) and tested within 24 h.

**Table 1.** Types of food sampled in Poland from which Enterobacterales species were isolated

	Type of food	Origin of the raw material	Number of samples tested
Cold-smoked fish			
	Salmon slices, loin, fillet and scraps	Norway	41
	Salmon fillet	Denmark	1
	Salmon	Scotland	1
	Halibut fillet	Norway	1
	Herring fillet	Norway	1
	Trout	Norway	1
Cold cuts			
	Smoked pork (ham, tenderloin, bacon, gammon, loin, neck and shoulder)	Poland	51
	Pork sausage	Poland	26
	Brawn	Poland	4
	White sausage	Poland	3
	Pork pate	Poland	3
	Blood sausage	Poland	2
	Pastrami	Poland	2
	Pork and chicken sausage	Poland	1
	Pork fat	Poland	1
	Steamed minced pork	Poland	1
	Veal and pork pate	Poland	1
	Smoked poultry tenderloin	Poland	1
Cheeses			
	Rennet goat cheese	Poland	14
	Rennet cow’s milk cheese	Poland	10
	Cow’s milk cottage cheese	Poland	2
	Oscypek smoked sheep cheese	Poland	5
	Goat cheese curd	Poland	3
	Ricotta-type goat cheese	Poland	3
	Smoked goat cheese	Poland	3
	Bundz unsmoked sheep cheese	Poland	3
	Halloumi goat cheese	Poland	1
	Halloumi cow’s milk cheese	Poland	1
	Brined goat cheese	Poland	1
	Grilled goat cheese	Poland	1
	Blue goat cheese	Poland	1
	Feta goat cheese	Poland	1
	Mozzarella-type cow’s milk cheese	Poland	1
	Paneer-type cow’s milk cheese	Poland	1
	Feta cow’s milk cheese	Poland	1

### Bacterial isolation and identification

The samples were prepared for microbiological examination according to the part of the International Organization for Standardization (ISO) 6887 standard appropriate to the product concerned (11, 12, 13, 14). Bacteria of the Enterobacterales order were detected using the method stipulated in the ISO 21528-1:2017 standard (10). Briefly, 10 ± 0.1 g of sample was pre-enriched with 90 mL of non-selective Buffered Peptone Water (Oxoid, Basingstoke, UK) at 37 ± 1°C for 18 ± 2 h. Afterwards, one loopful of enriched BPW was streaked on selective Violet Red Bile Glucose (VRBG) agar (Bio-Rad, Hercules, CA, USA) and incubated at 37 ± 1°C for 24 ± 2 h. From each sample with presumptive Enterobacterales after incubation, from one to five characteristic but morphologically different bacterial colonies (pink, red or purple with or without precipitation haloes) were sub-cultured on nutrient agar (Bio-Rad) and confirmed biochemically for fermentation of glucose and the presence of oxidase. Then, species identification of the isolated Enterobacterales was carried out based on their protein profile using the matrix-assisted laser desorption/ionisation–time-of-flight mass spectrometry (MALDI-TOF) technique and the Biotyper system (Bruker Daltonics, Bremen, Germany).

### Antimicrobial resistance testing

The antimicrobial susceptibility of Enterobacterales isolates was tested by the minimal inhibitory concentration (MIC) method with the EUVSEC plate (Trek Diagnostic System, East Grinstead, UK) containing a panel of 14 antimicrobials, including those used for treatment of human infections. The antimicrobials, dilution ranges and cut-off values used for MIC interpretation are described in Table 2.

**Table 2.** Antimicrobials, dilution ranges and cut-off values used for minimum inhibitory concentration interpretation of tested Enterobacterales

Antimicrobial class	Antimicrobial	Dilution range (mg/L)	Cut-off value (mg/L)
	Ampicillin	1–64	8
Beta-lactams	Cefotaxime	0.25–4	0.25
(penicillins, cephalosporins and carbapenems)	Cephtazidime	0.50–8	1
	Meropenem	0.03–16	0.06
Phenicols	Chloramphenicol	8–128	16
Quinolones	Nalidixic acid	4–128	8
Fluoroquinolones	Ciprofloxacin	0.015–8	0.06
Aminoglycosides	Gentamicin	0.50–32	2
Macrolides	Azithromycin	2–64	16
Polymyxins	Colistin	1–16	2
Tetracyclines	Tetracycline	2–64	8
Glycylcyclines	Tigecycline	0.25–8	0.50
Folate pathway inhibitors	Sulphamethoxazole	8–1024	64
	Trimethoprim	0.25–32	2

The isolates were sub-cultured twice on nutrient agar at 37 ± 1°C for 24 ± 2 h. A bacterial suspension was prepared in Sensitre Sterile Water (Thermo Fisher Scientific, Waltham, MA, USA) with a density equivalent to 0.5 McFarland standard, and 10 μL of the suspension was transferred to 11 mL of Sensitre Cation Adjusted Mueller–Hinton Broth (Thermo Fisher Scientific). Fifty microlitres of the bacterial suspension was then applied to each well of a microtitre plate containing the specified concentration of antimicrobial agents, incubated at 35 ± 1°C for 18 ± 2 h and then read using the Sensitre Vision System (Trek Diagnostic System). *Escherichia coli* ATCC (American Type Culture Collection) 25922 was used in each analysis as the antimicrobial quality control. The cut-off values used for the interpretation of the MIC results were in accordance with the European Committee on Antimicrobial Susceptibility Testing (EUCAST) and the European Union Reference Laboratory for Antimicrobial Resistance recommendations (6, 7). Multidrug resistance of the tested isolates was defined as resistance to at least three classes of the antimicrobials used in the study (18).

## Results

### Prevalence and identification of Enterobacterales

Among 194 products tested, 159 (82.0%) were positive for Enterobacterales bacteria. The highest percentage of positive samples was observed in cheeses (49 out of 52 samples; 94.2%), mainly in those made from unpasteurised goat’s milk (27 out of 28 samples; 96.4%). Other food samples were also often positive for these microorganisms (Table 3). A total of 226 Enterobacterales isolates were obtained from the 159 positive samples, nearly half coming from cold cuts (101 isolates), 80 isolates from cheeses and 45 isolates from cold-smoked fish. The detailed results of these analyses are shown in Table 3.

**Table 3.** Presence of Enterobacterales in retail food tested

Type of food		Number (%) of samples tested		Number (%) of positive samples		Number of isolates	
Cold cuts		96 (49.5)		74 (77.1)		101	
Cold-smoked fish		46 (23.7)		36 (78.3)		45	
Cheeses		52 (26.8)		49 (94.2)		80	
goat cheese		28 (14.4)		27 (96.4)		49	
cow’s milk cheese		16 (8.3)		15 (93.8)		20	
sheep cheese		8 (4.1)		7 (87.5)		11	
Total		194 (100)		159 (82.0)		226	

**Fig. 1.**
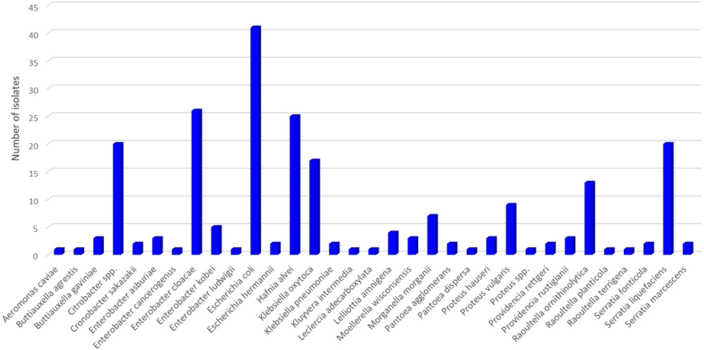
Prevalence and species of Enterobacterales bacteria isolated from tested retail food

Based on species identification using the MALDI-TOF technique, the largest genus group among the 226 Enterobacterales isolates was *Escherichia* (43 isolates; 19.0%), the next-largest was *Enterobacter* (36; 15.9%); *Hafnia* (25; 11.1%) was a smaller group; *Serratia* (24; 10.6%) was nearly equal to *Hafnia*; and *Citrobacter* (20; 8.8%), *Klebsiella* (19; 8.4%), *Raoultella* (15; 6.6%) and *Proteus* (13; 5.8%) were the remaining significant genera (Fig. 1). The most frequently isolated microorganisms were *Escherichia coli* (n = 41), *Enterobacter cloacae* (n = 26), *Hafnia alvei* (n = 25), *Citrobacter* spp. (including *C. braakii, C. freundii* and *C. gillenii*; n = 20), *Serratia liquefaciens* (n = 20) and *Klebsiella oxytoca* (n = 17) (Fig. 1). The remaining bacterial species were identified in fewer than 15 instances each (Fig. 1).

Analysing the types of food tested for Enterobacterales prevalence, it was observed that the same microorganisms were most often isolated from cold cuts and cold-smoked fish: 13 *H. alvei* isolates were taken from meat and 11 from fish, 13 *E. cloacae* isolates were contained in meat and 6 were in fish, 10 *S. liquefaciens* isolates were revealed from meat and 8 from fish, and 9 *Citrobacter* spp. originated from meat and 4 from fish. The species accounting for the largest proportions of Enterobacterales strains isolated from cheeses were *E. coli* (32 isolates), *K. oxytoca* (9 isolates), *Citrobacter* spp. (7 isolates), *E. cloacae* (7 isolates) and *Raoultella ornithinolytica* (6 isolates). The microorganisms in goat cheeses were mainly *E. coli* (n = 21), *Enterobacter* spp. (n = 7), *K. oxytoca* (n = 6) and *R. ornithinolytica* (n = 5). Isolates totalling 11 out of 20 from cheeses made from raw cow’s milk were recognised as *E. coli*, and the remaining single isolates belonged to various species of Enterobacterales bacteria. In cheeses made from unpasteurised sheep’s milk, 5 isolates belonging to the *Citrobacter* genus, and 2 each to the *Proteus, Enterobacter* and *Klebsiella* genera were identified.

### Antimicrobial resistance among Enterobacterales

**Fig. 2.**
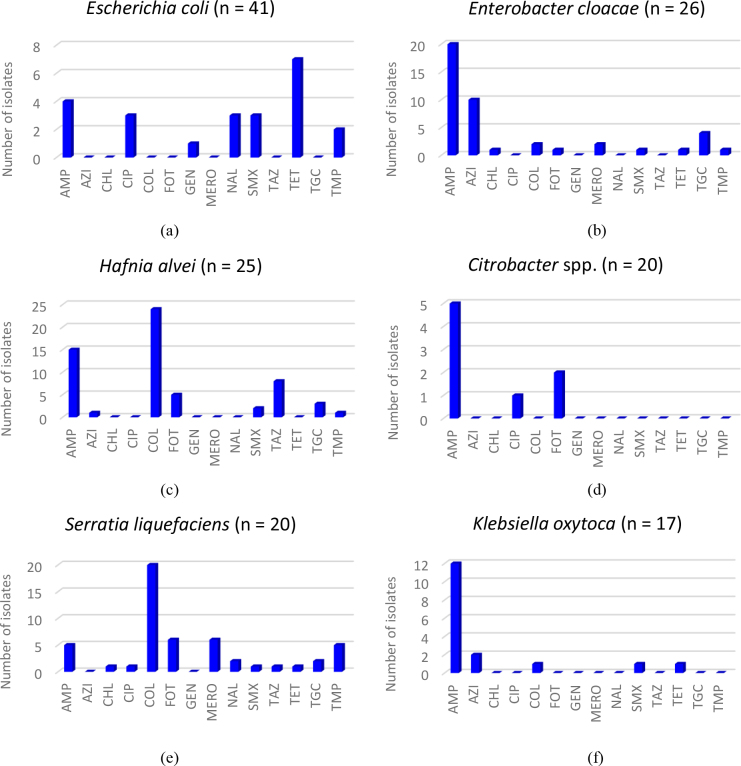
Antimicrobial resistance among selected species of Enterobacterales: (a) *Escherichia coli*; (b) *Enterobacter cloacae*; (c) *Hafnia alvei*; (d) *Citrobacter* spp.; (e) *Serratia liquefaciens*; (f) *Klebsiella oxytoca*; AMP – ampicillin; AZI – azithromycin; CHL – chloramphenicol; CIP – ciprofloxacin; COL – colistin; FOT – cefotaxime; GEN – gentamicin; MERO – meropenem; NAL – nalidixic acid; SMX – sulphamethoxazole; TAZ – cephtazidime; TET – tetracycline; TGC – tigecycline; TMP – trimethoprim

The most frequently isolated Enterobacterales bacteria, *i.e. E. coli, E. cloacae, H. alvei, Citrobacter* spp., *S. liquefaciens* and *K. oxytoca* were tested for antimicrobial resistance (Fig. 2). It was observed that 7 out of 41 (17.1%) *E. coli* strains were resistant to tetracycline and fewer strains were resistant to ampicillin, ciprofloxacin, nalidixic acid, sulphamethoxazole, trimethoprim and gentamicin (Fig. 2a). *E. cloacae* isolates were mostly resistant to ampicillin (20 out of 26; 76.9%) and azithromycin (10 out of 26; 38.5%) (Fig. 2b). All but one *H. alvei* isolate (24 out of 25; 96.0%) were resistant to colistin, and most of them were resistant to ampicillin (15 out of 25; 60.0%) (Fig. 2c). Interestingly, the majority of cephtazidime-resistant Enterobacterales (88.9%) were identified as *H. alvei*. All strains of *S. liquefaciens* (n = 20) were resistant to colistin; some of these isolates also showed resistance to cefotaxime, meropenem, ampicillin and trimethoprim (Fig. 2e). The majority of *K. oxytoca* isolates (12 out of 17; 70.6%) were resistant to ampicillin (Fig. 2f), whereas only 5 *Citrobacter* isolates were ampicillin resistant, and single isolates also revealed resistance to cefotaxime and ciprofloxacin (Fig. 2d).

Among the six most frequently isolated Enterobacterales species, 20 strains (8.8% of all 226 Enterobacterales isolates) were MDR, and among them were strains even resistant to six classes (Table 4). It was observed that *S. liquefaciens* (5; 25.0%), *H. alvei* (5; 20.0%), *E. cloacae* (5; 19.2%) and *E. coli* (4; 9.8%) showed MDR patterns. However, only one *K. oxytoca* isolate and none of the *Citrobacter* isolates were MDR. Interestingly, no such strains were isolated from cold-smoked fish (Table 4).

**Table 4.** Antimicrobial multidrug resistance profiles among selected Enterobacterales isolates (n = 20)

Isolate species	Origin of the isolate	Antimicrobial resistance profile	Number of antimicrobial classes
*Hafnia alvei*	Cold cuts	AMP-COL-SMX	3
*Hafnia alvei*	Cold cuts	AMP-TAZ-COL-TGC	3
*Hafnia alvei*	Cold cuts	AZI-FOT-COL-TGC	4
*Hafnia alvei*	Cold cuts	AMP-COL-SMX	3
*Hafnia alvei*	Cheeses	AMP-COL-TGC	3
*Enterobacter cloacae*	Cold cuts	AMP-AZI-COL	3
*Enterobacter cloacae*	Cold cuts	AMP-AZI-TGC	3
*Enterobacter cloacae*	Cheeses	AMP-AZI-TGC	3
*Enterobacter cloacae*	Cheeses	AMP-CHL-COL-TET-TGC-SMX-TMP	6
*Enterobacter cloacae*	Cheeses	AMP-AZI-TGC	3
*Escherichia coli*	Cold cuts	AMP-CIP-NAL-TET	4
*Escherichia coli*	Cold cuts	AMP-CIP-NAL-TET	4
*Escherichia coli*	Cold cuts	AMP-CIP-NAL-SMX-TMP	4
*Escherichia coli*	Cheeses	AMP-TET-SMX-TMP	3
*Serratia liquefaciens*	Cold cuts	FOT-TAZ-MERO-CIP-COL-NAL	4
*Serratia liquefaciens*	Cold cuts	MERO-COL-TMP	3
*Serratia liquefaciens*	Cheeses	FOT-MERO-COL-TMP	3
*Serratia liquefaciens*	Cheeses	AMP-MERO-CHL-COL-TET-TGC-SMX-TMP	6
*Serratia liquefaciens*	Cold cuts	AMP-FOT-COL-TMP	3
*Klebsiella oxytoca*	Cheeses	AMP-AZI-SMX	3

1 AMP – ampicillin; COL – colistin; SMX – sulphamethoxazole; TAZ – cephtazidime; TGC – tigecycline; AZI – azithromycin; FOT – cefotaxime; CHL – chloramphenicol; TET – tetracycline; TMP – trimethoprim; CIP – ciprofloxacin; NAL – nalidixic acid; MERO – meropenem

## Discussion

Food may be a source of bacterial pathogens, including antimicrobial resistant strains which pose a serious threat to consumer health. For this reason, studies on the prevalence of potentially pathogenic microorganisms in different foods are performed in many countries. In the present study, the prevalence and antimicrobial resistance of Enterobacterales bacteria isolated from food products of animal origin available in retail in Poland were described. A high prevalence of Enterobacterales was observed in all tested samples. These results are similar to those reported by other authors, where *Enterobacteriaceae* were detected at a level of 87.2% in Portuguese deli meats and in 68.0%–100% of Egyptian fish samples, with the percentage depending on the species of fish tested (2, 28). The dairy product results of previous research are also consistent with our results: in Egyptian raw milk *Enterobacteriaceae* were identified in 84% of samples, and in Spanish unpasteurised San Simón cheese they were found in 96.1% (31, 34), which confirms that cheeses produced from raw milk may be contaminated by *Enterobacteriaceae* (20). On the other hand, lower ranges of *Enterobacteriaceae* prevalence were observed by Sobeih *et al*. (31) in Egyptian yogurt (32%–40%) and ice cream (20%–64%).

In the current investigation, *E. coli, E. cloacae, H. alvei, Citrobacter* spp., *S. liquefaciens* and *K. oxytoca* were the most frequently isolated bacteria. Similar results were obtained by other authors who observed a significant contamination of different food products with such bacteria (19, 31, 32). In the present study it was also noted that the presence of specific Enterobacterales microorganisms was related to the type of samples tested. *H. alvei, E. cloacae*, and *S. liquefaciens* were most frequently detected both in cold cuts and cold-smoked fish, whereas the majority of bacterial isolates from cheeses were recognised as *E. coli*. A large species diversity of bacteria isolated from various products similar to that observed in this study was also described by other authors. A previous study conducted in Poland (32) showed that *Enterobacter* spp., *Proteus* spp., *Hafnia* spp., *Serratia* spp., *Klebsiella* spp. and *E. coli* were the most frequently detected microorganisms in cold cuts. Furthermore, *E. coli, M. morganii, K. oxytoca* and *K. pneumoniae* were isolated from deli meat in Portugal (2). In addition, Jansen *et al*. (17) found *E. coli* in meat products imported into the European Union, and in the large-scale studies of Schwaiger *et al*. (30), *Enterobacter, Citrobacter, Serratia* and *Klebsiella* were the most common microbial contamination identified in chicken and pork. However, these results are difficult to compare with our findings, because those studies were related to raw meat and not the retail meat products which were analysed in the current investigation. According to Mladenović *et al*. (20, 21), *K. oxytoca, K. pneumoniae, K. ornithinolytica* and *E. coli* were the most frequently identified bacterial species in raw milk cheese. Trmčić *et al*. (35) indicated that the milk type used for cheese production was also significantly associated with the species of *Enterobacteriaceae* detected in the finished product, which is consistent with our results. This may be related to the microbiological quality of the raw material used in the food production, the processing and technological processes and the distribution and storage conditions before consumption. Mostly dissonant species findings were presented in the investigations conducted on dairy products in Egypt, in which the most often isolated microorganisms from raw milk samples were *Hafnia alive* (31.0%), *S. liquefaciens* (25.0%) and *K. pneumonia* (15.5%), whereas *E. coli* was the predominant microorganism identified in yoghurt (44.4%) (31). In another study, 221 isolates from San Simón cheese in Spain were mainly identified as *K. oxytoca, H. alvei, E. coli, E. cloacae* and *C. freundii* (34). Some literature data which are similar to our current results indicate noticeable associations between the type of food samples and their contamination with specific Enterobacterales species. The *Proteus, Hafnia, Escherichia* and *Enterobacter* genera are usually associated with mammalian gastrointestinal systems, and their occurrence in meat, cold cuts or dairy products may be the result of food cross-contamination (16, 19, 31). However, the sources of microbiological contamination of food of animal origin intended for direct human consumption are usually not clear and may be related to contamination at various stages of food production.

The six most frequent Enterobacterales bacteria identified in the current study were then analysed for resistance to antimicrobials. It was shown that *E. coli* was mainly resistant to tetracycline, but also to a lesser extent to ampicillin, ciprofloxacin, nalidixic acid, sulphamethoxazole, trimethoprim and gentamicin. Similar results were obtained by the authors of other studies, which showed that *E. coli* isolates very often were resistant to many classes of antibacterial agents. In the study of Ryu *et al*. (27), resistance to tetracycline (30.7%), streptomycin (12.8%), cephalothin (11.7%), ampicillin (6.7%), trimethoprim/sulphamethoxazole (6.7%) and nalidixic acid (5.6%) was found in *E. coli* strains isolated from fish and seafood in Korea. The majority of isolates identified in our study as *E. cloacae* were resistant to ampicillin and azithromycin, whereas *K. oxytoca* was mainly resistant to ampicillin. It was consistent with the observations reported in other studies (2, 19, 39). However, in the investigation of Schwaiger *et al*. (30), *E. cloacae* isolated from meat products were mainly colistin-resistant (45.5%), and *K. oxytoca* and *Citrobacter* isolates were resistant to many antimicrobial agents from the β-lactam and aminoglycoside classes. In the present investigation, low resistance (especially to ampicillin, cefotaxime and ciprofloxacin) was observed among *Citrobacter* isolates, while a high level of resistance to colistin was identified in *S. liquefaciens* and in *H. alvei*, which were also resistant to β-lactams. These observations were in line with the results reported by Szewczyk *et al*. (32) and Mladenović *et al*. (19).

Our findings also showed that 8.8% of isolates tested were MDR, which is a lower percentage than those reported by other authors. Amador *et al*. (2, 3) observed MDR phenotypes among isolates from delicatessen meat (35.9%) and cheese (31.4%). In that study, MDR was often associated with *E. coli* and *S. enterica*. Multidrug resistant isolates of *Enterobacter, Citrobacter* and *Klebsiella* species were also described by Chauhan *et al*. (4) and Fakruddin *et al*. (8) in various food samples, including raw milk and dairy milk products. On the other hand, Schwaiger *et al*. (30) and Uzeh *et al*. (36) identified MDR strains in *Enterobacter* spp., *Serratia* spp., *Klebsiella* spp. and *Citrobacte*r spp. isolated from chicken meat.

## Conclusion

The results of the present investigation indicate that retail food can be contaminated with various species of Enterobacterales, including isolates resistant to commonly used antimicrobial agents. Some bacterial species diversity in different food types was identified. The obtained results revealed that most Enterobacterales isolates showed resistance to the ampicillin and colistin, and to a lesser extent to cefotaxime and ceftazidime of the third-generation cephalosporins, and meropenem belonging to carbapenems. The significant resistance to antimicrobial agents, including MDR strains, may be the result of the common use of antibiotics in agriculture and animal husbandry. For this reason, it is necessary to monitor microbial resistance in the environment, including bacteria belonging to the Enterobacterales order, which are commonly isolated from food. Enterobacterales have public health and economic importance, and because they are considered indicatory microorganisms for the microbiological quality of food and the hygiene standards of its production, their presence in large numbers may suggest improper processing or post-processing contamination of food.
